# 3D synergistic tumor-liver analysis further improves the efficacy prediction in hepatocellular carcinoma: a multi-center study

**DOI:** 10.1186/s12885-025-13501-9

**Published:** 2025-01-21

**Authors:** Yurong Jiang, Jiawei Zhang, Zhaochen Liu, Jinxiong Zhang, Xiangrong Yu, Danyan Lin, Dandan Dong, Mingyue Cai, Chongyang Duan, Shuyi Liu, Wenhui Wang, Yuan Chen, Qiyang Li, Weiguo Xu, Meiyan Huang, Sirui Fu

**Affiliations:** 1 Department of Radiology, Zhuhai Clinical Medical College of Jinan University (Zhuhai People’s Hospital, The Affiliated Hospital of Beijing Institute of Technology), No. 79 Kangning Road, Zhuhai, 519000 Guangdong Province China; 2 Zhuhai Interventional Medical Center, Zhuhai People’s Hospital, The Affiliated Hospital of Beijing Institute of Technology, Zhuhai Clinical Medical College of Jinan University, No. 79 Kangning Road, Zhuhai, 519000 Guangdong Province China; 3 Zhuhai Engineering Technology Research Center of Intelligent Medical Imaging, Zhuhai People’s Hospital, The Affiliated Hospital of Beijing Institute of Technology, Zhuhai Clinical Medical College of Jinan University, No. 79 Kangning Road, Zhuhai, 519000 Guangdong Province China; 4 School of Biomedical Engineering, Southern Medical University, No. 1023-1063 Shatai Road, Guangzhou, 510515 Guangdong Province China; 5 Department of Hepatobiliary and Pancreatic Surgery, the First Affiliated Hospital of Zhengzhou University, No. 1 Jianshe East Road, Zhengzhou, 450000 Henan Province China; 6 Department of Interventional Radiology, School of Medicine, Guangzhou First People’s Hospital, South China University of Technology, Guangzhou, China; 7 Department of Minimally Invasive Interventional Radiology, the Second Affiliated Hospital of Guangzhou Medical University, Changgang East Road, Guangzhou, 510000 Guangdong Province China; 8 Department of Biostatistics, School of Public Health, Southern Medical University, No. 1023-1063 Shatai Road, Guangzhou, 510515 Guangdong Province China; 9 Department of Clinical Medicine, First Clinical Medical College, Southern Medical University, No. 1023-1063 Shatai Road, Guangzhou, 510515 Guangdong Province China; 10 Department of Medical Imaging, First Clinical Medical College, Southern Medical University, No. 1023-1063 Shatai Road, Guangzhou, 510515 Guangdong Province China; 11 Department of Interventional Treatment, Zhongshan City People’s Hospital, No. 2, Sunwen East Road, Zhongshan, 528400 Guangdong Province China; 12 Department of Interventional Radiology, The Second Clinical Medical College, Shenzhen People’s Hospital, The Second Clinical Medical College, Jinan University; The First Affiliated Hospital, Southern University of Sciences and Technology, No. 1017, Dongmen North Road, Shenzhen, 518020 Guangdong Province China; 13 Guangdong Provincial Key Laboratory of Medical Image Processing, Southern Medical University, No. 1023-1063 Shatai Road, Guangzhou, 510515 Guangdong Province China; 14 Guangdong Province Engineering Laboratory for Medical Imaging and Diagnostic Technology, Southern Medical University, No. 1023-1063 Shatai Road, Guangzhou, 510515 Guangdong Province China

**Keywords:** Hepatocellular carcinoma, Tumor, Liver parenchyma, 3D assessment, Automatic segmentation, Transcatheter arterial chemoembolization, Liver resection, Treatment efficacy prediction, Progression-free survival, Visualized model

## Abstract

**Background:**

Besides tumorous information, synergistic liver parenchyma assessments may provide additional insights into the prognosis of hepatocellular carcinoma (HCC). This study aimed to investigate whether 3D synergistic tumor-liver analysis could improve the prediction accuracy for HCC prognosis.

**Methods:**

A total of 422 HCC patients from six centers were included. Datasets were divided into training and external validation datasets. Besides tumor, we also performed automatic 3D assessment of liver parenchyma by extracting morphological and high-dimensional data, respectively. Subsequently, we constructed a tumor model, a tumor-liver model, a clinical model and an integrated model combining information from clinical factors, tumor and liver parenchyma. Their discrimination and calibration were compared to determine the optimal model. Subgroup analysis was conducted to test the robustness, and survival analysis was conducted to identify high- and low-risk populations.

**Results:**

The tumor-liver model was superior to the tumor model in terms of both discrimination (training dataset: 0.747 vs. 0.722; validation dataset: 0.719 vs. 0.683) and calibration. Moreover, the integrated model was superior to the clinical model and tumor-liver model, particularly in discrimination (training dataset: 0.765 vs. 0.695 vs. 0.747; validation dataset: 0.739 vs. 0.628 vs. 0.719). The AUC of the integrated model was not influenced by AFP level, BCLC stage, Child–Pugh grade, and treatment style in training (6 months *p* value: 0.245–0.452; 12 months *p* value: 0.357–0.845) and validation (6 months *p* value: 0.294–0.638; 12 months *p* value: 0.365–0.937) datasets. With a risk score of 1.06, high- and low-risk populations demonstrated significant difference for progression-free survival (*p* < 0.001 in both datasets).

**Conclusions:**

Combined with clinical factors, 3D synergistic tumor-liver assessment improved the efficacy prediction of HCC.

**Supplementary Information:**

The online version contains supplementary material available at 10.1186/s12885-025-13501-9.

## Background

Primary liver cancer is the fifth most common cancer worldwide and second leading cause of cancer-related deaths, with hepatocellular carcinoma (HCC) accounting for 75 − 86% of cases [1, 2]. For HCC without portal vein thrombus or extrahepatic metastasis, liver resection and transcatheter arterial chemoembolization (TACE) are the first-line treatments recommended by guidelines [3, 4]. Models predicting the efficacy of liver resection and TACE have consistently been a focus in HCC studies to enhance scientific decision-making. Information for efficacy prediction can be achieved through multiple ways. One is clinical information, such as alpha-fetoprotein, Barcelona Clinic Liver Cancer (BCLC) stage, etc. The other is data extracted from CT or MRI images [5–8]. Currently, image information mainly focused on tumor area, however, HCC metastasis might also be influenced by the liver parenchyma, which may have stimulative or restrictive functions, such as the occurrence of liver fibrosis or capsule formation [9, 10]. Thus, evaluation of the liver parenchyma may provide additional information for HCC prognosis.

Recently, more and more researchers used information from liver parenchyma for HCC prognosis prediction, such as fibrosis markers and liver stiffness [11–13]. These studies prove that using liver parenchyma information to predict HCC prognosis is feasible and reasonable. However, more effort should be made to extract in-depth information from the liver parenchyma. On the one hand, 2D manual method should be improved into 3D automatic assessment. On the other hand, rather than single parameter, multi-dimensional data within and beyond traditional visual system should be synchronously evaluated. To address this aspect, we previously developed a nnU-net based 3D assessment for liver (detailed in Supplementary Text 1), which evaluated both morphological and high-denominational change under liver cirrhosis [14]. Based on these reasons, developing automatic 3D assessment for liver can contribute significantly to predicting HCC prognosis.

**Fig. 1:**
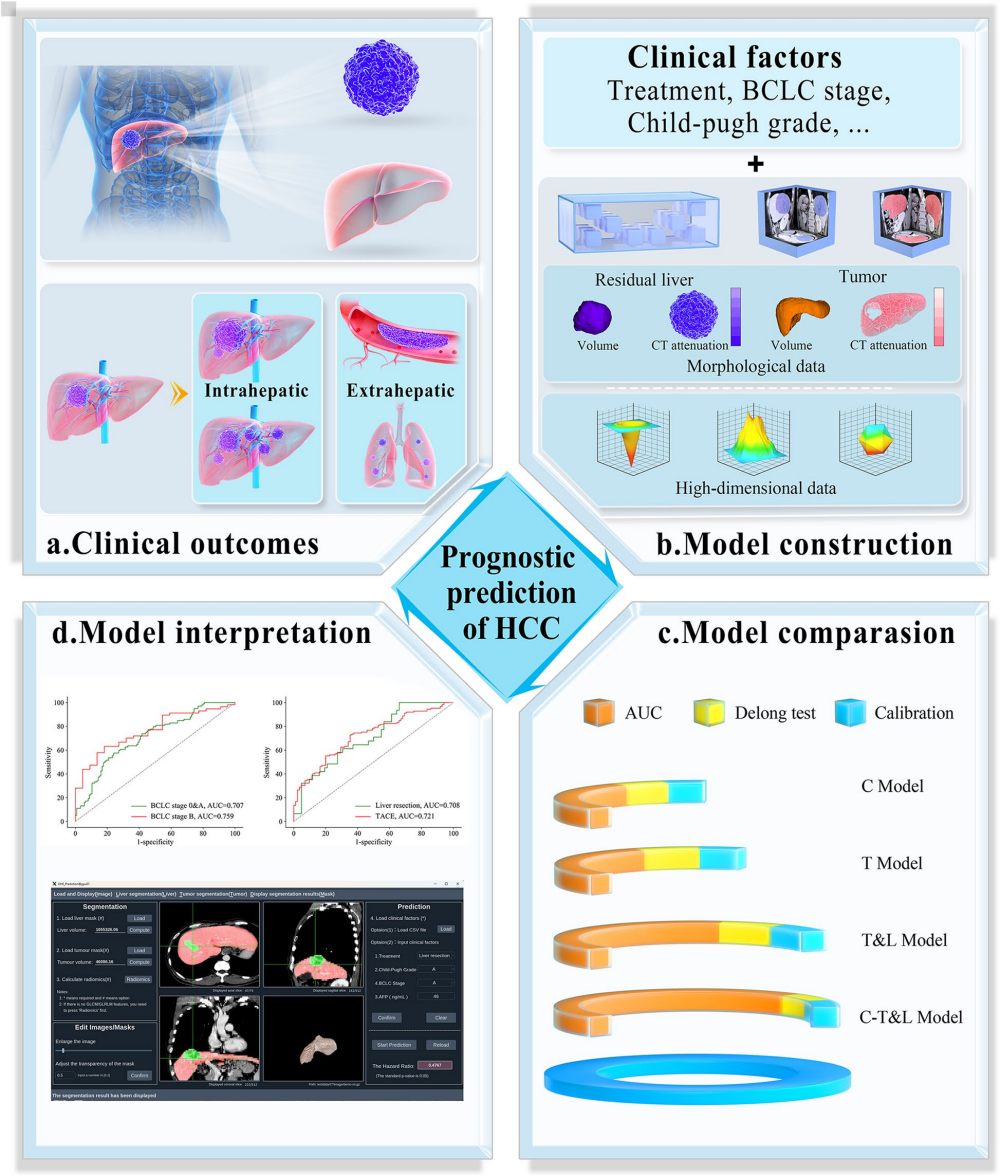
Study design. **a**. This study aims to construct models for predicting the treatment efficacy for HCC. **b**. In addition to clinical factors, candidate indicators include tumor and liver information obtained by automatic 3D assessment. **c**. The tumor model (T Model) is compared with the tumor-liver model (T&L Model) to demonstrate the necessity of liver information. We then compare the clinical model (T Model), T&L Model, and integrate model (C-T&L model) to identify the optimal model. **d**. Subgroup and survival analyses are performed using the constructed applet. HCC: hepatocellular carcinoma; AUC: area under the curve

Considering the physiopathologic mechanism of HCC, in addition to traditional clinical indicators, we performed this study including 3D synergistic tumor-liver to improve the efficacy prediction of HCC (Fig. 1).

## Methods

### Patient selection

**Fig. 2:**
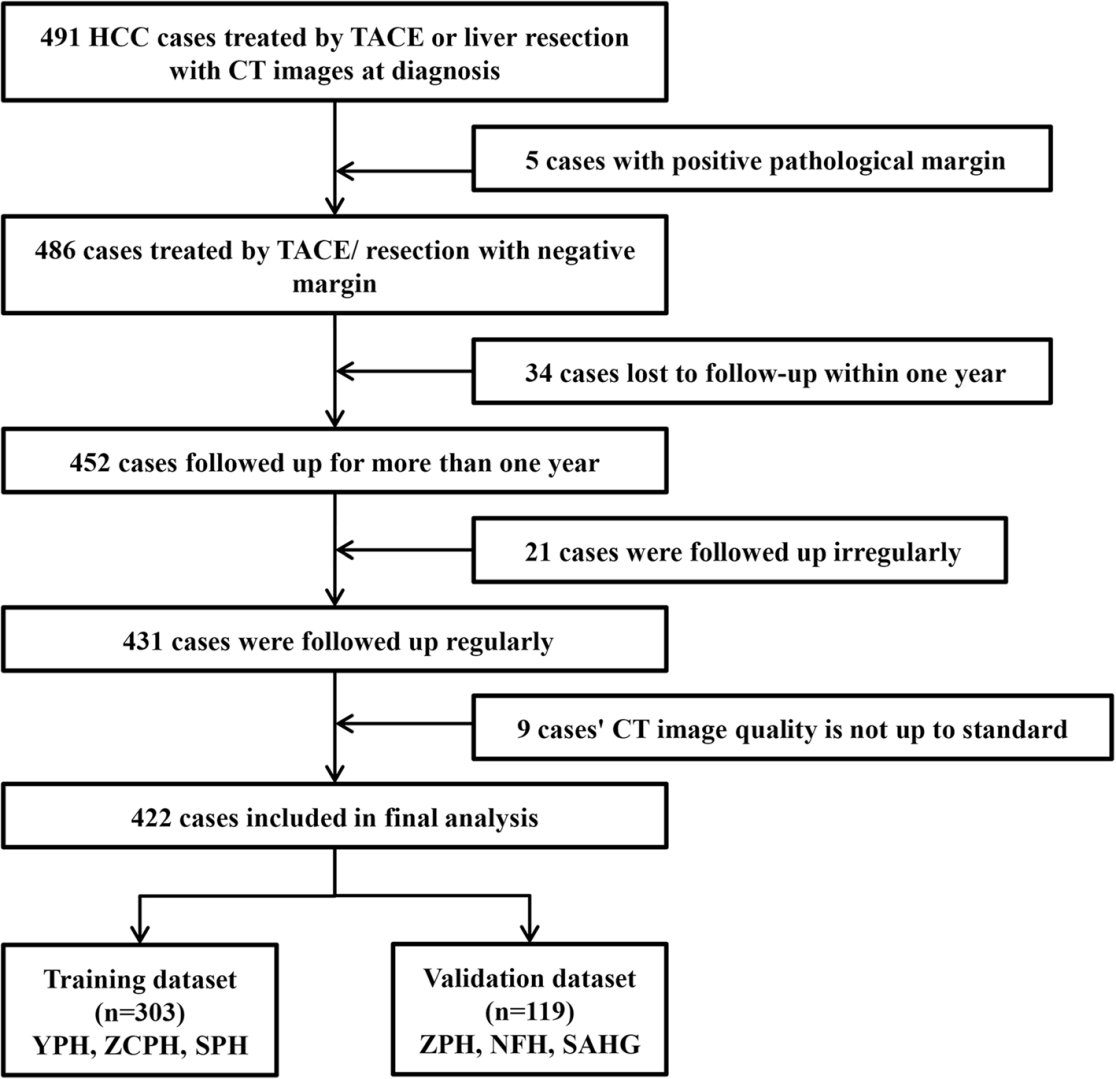
Inclusion and exclusion flowchart. The inclusion and exclusion flowchart showing patient selection for this study. A total of 491 patients from six hospitals are preliminarily identified. After applying the inclusion and exclusion criteria, 422 patients are identified and divided into training (*n* = 303) and external validation (*n* = 119) datasets

Patients diagnosed with HCC between July 2006 and December 2020 were screened. Data were collected from six hospitals in China: Zhuhai People’s Hospital (ZPH), Yangjiang People’s Hospital (YPH), Zhongshan City People’s Hospital (ZCPH), Shenzhen People’s Hospital (SPH), Nanfang Hospital (NFH), and The Second Affiliated Hospital of Guangzhou Medical University (SAHG). The end date of follow-up was August 2022. Inclusion criteria comprised: (1) clinical or pathological diagnosis of HCC following guidelines [3, 4, 15]; (2) enhanced CT performed within 1 month before initial treatments; (3) initial treated by liver resection or TACE following guidelines, and (4) identification of progressive disease (PD) during treatment or regular follow-up without PD for at least 12 months unless death occurred. Exclusion criteria were: (1) postoperative pathology of patients undergoing hepatectomy showed positive margins, (2) patients without PD who had a follow-up less than 12 months, (3) irregular follow-up, and (4) unqualified CT image, such as image artifact interference. After applying the inclusion and exclusion criteria, 422 patients were included in this study (Fig. 2).

All the procedures were in accordance with the ethical standards of the responsible committee on human experimentation (institutional and national) and with the Helsinki Declaration of 1975, as revised in 2008. Informed consent for participation was waived because the patients’ data were collected retrospectively. All patients’ data were anonymized before analysis.

### Treatment and follow-up

Initial treatment included liver resection or TACE, a decision made by a multidisciplinary team relying on established guidelines [3, 4, 15, 16], considering tumor characteristics, liver function, and patients’ treatment intention. Patients who underwent liver resection had macroscopic tumors removed during surgery, and postoperative pathology confirmed a negative surgical margin. TACE involved super-selective embolization of the hepatic artery with lipiodol and chemotherapeutic drugs under digital subtraction angiography guidance, with the surgeon adhering to operation and treatment standards.

Follow-up assessments occurred every 4–6 weeks for the first year, extending to 3–6 months if the patient showed no signs of PD. The follow-ups included laboratory tests, general imaging examinations (including chest radiography and abdominal ultrasonography), and further CT or MRI if extrahepatic metastasis was suspected.

### Outcomes

The primary outcome of this study was PD within 12 months. The secondary outcome was progression-free survival (PFS), defined as the period from initial treatment to the occurrence of PD. PD was confirmed by CT and MRI following the modified Response Evaluation Criteria in Solid Tumors (mRECIST) criteria for HCC [17].

### Clinical factors

The clinical indicators are listed in Table 1. These clinical factors included the general patient characteristics and status (such as age, sex, hepatitis B infection, and Child–Pugh grade etc.), tumor burden (such as BCLC stage and AFP level etc.) and initial treatments (liver resection or TACE). Additionally, the neutrophil-to-lymphocyte ratio (NLR) was included in the analysis [18].

**Table 1:** Baseline demographics of patients

Clinical factors	Training dataset (*n* = 303)	Validation dataset (*n* = 119)	*p*-value
**Age** (years)	57.45 ± 12.20	56.75 ± 12.20	0.866
**Sex** (n)			0.028*
Male	273	98	
Female	30	21	
**Hepatitis B** (n)			< 0.001*
Negative	42	40	
Positive	261	79	
^**Child–Pugh grade** (n)^			0.047*
A	267	96	
B	36	23	
^**BCLC stage** (n)^			0.679
0	19	5	
A	205	84	
B	79	30	
**Treatments** (n)			0.441
Liver resection	93	32	
TACE	210	87	
**AFP level** (n)			0.995
< 20 ng/mL	131	52	
20–400 ng/mL	75	29	
> 400 ng/mL	97	38	
**ALT** (U/L)	38.00 (26.00, 56.00)	31.00 (22.00, 47.00)	0.003*
**NLR**	2.29 (1.54, 3.72)	2.21 (1.33, 3.36)	0.167

Normally distributed factors are expressed using means ± standard deviations; non-normally distributed factors are expressed as medians (interquartile ranges)

*ALT* alanine aminotransferase, *NLR* neutrophil to lymphocyte ratio, *AFP* alpha-fetoprotein, *BCLC* Barcelona Clinic Liver Cancer

^*^ *p* < 0.050

Notably, the number of tumors and the maximum diameter of tumors were classified as tumor-related features and were not included in clinical factors. In addition, compared with the diameter of the tumor, the volume of the tumor can more appropriately reflect the size of the tumor, so the volume of the tumor is included in the analysis, but not the diameter of the tumor. In addition, to avoid collinearity, the number of tumors used to stage BCLC and the indicators used to calculate Child–Pugh grade (including albumin and total bilirubin) were not included separately.

### CT data acquisition

The CT parameters for the participating hospitals are listed in Supplementary Table 1. Considering that HCC had a clearer boundary in the portal phase than other phases [19], in order to ensure the accuracy of segmentation, we used the portal phase as the analytical data. When multiple lesions were present, we identified the target lesion based on the longest diameter and suitability according to the modified Response Evaluation Criteria in Solid Tumors (mRECIST) [17].

### 3D segmentation of tumor and liver parenchyma

Firstly, we semiautomatically segmented the targeted lesion according to the mRECIST criteria. After automatic segmentation by nnU-Net, the results were confirmed by two radiologists (Yurong Jiang with 5 and Dandan Dong with 20 years of experience), with necessary manual corrections performed. Secondly, we automatically segmented the entire liver by nnU-Net, encompassing the targeted tumor. Then the targeted tumor was removed to achieve the region of liver parenchyma beyond targeted tumor.

### Data extraction for tumor and liver parenchyma

**Fig. 3:**
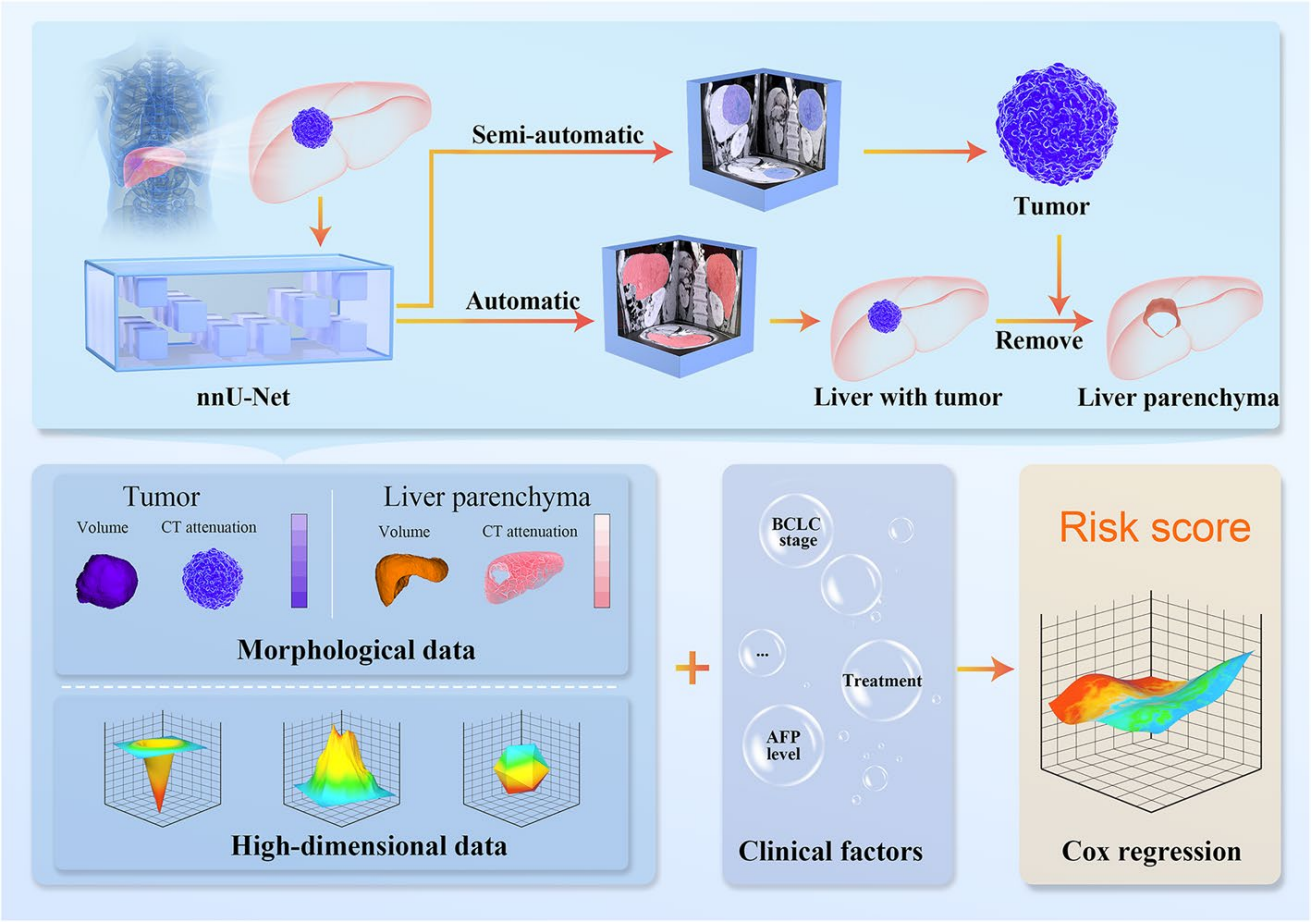
Workflow for model construction. After automatic segmentation using nnU-Net, tumor, and liver indicators are extracted, including morphological and high-dimensional data. Combined with clinical factors, tumor and liver indicators are used to construct a combined model with improved performance predicting treatment efficacy

For data extraction tumor and liver parenchyma, we measured both morphological and high-dimensional parameters. Morphological data included volume and CT attenuation data (Supplementary Table 2). To account for potential bias introduced by the scan protocol on CT attenuation, we calculated the quartile range and rate of change. Specifically, to address potential somatotype differences affecting liver parenchyma volume, we normalized the liver parenchyma volume using the maximum diameter of the portal vein (standardized liver parenchyma volume = liver parenchyma volume /portal vein diameter). For high-dimensional data, radiomics features were used to measure changes beyond the scope of human visual perception (Fig. 3).

### Statistical analyses

In this study, cases from six hospitals were divided into a training dataset (YPH, ZCPH, and SPH) and an external validation dataset (ZPH, NFH, and SAHG). Continuous variables are expressed as mean (standard deviation) or median (interquartile range) based on their distribution and compared using *t*-tests or Wilcoxon rank-sum tests. Categorical variables are expressed as percentages and compared using Pearson’s chi-square test or Fisher's exact test.

For model construction, we first used LASSO regression to screen all indicators. Second, we investigated the necessity of synergistic tumor and liver parenchyma indicators. Cox regression was employed to construct a tumor model (T Model) and a tumor-liver parenchyma model (T&L Model) using the indicators selected by LASSO regression. Subsequently, we compared the discrimination and calibration abilities of these models. Third, we explored the necessity of combining clinical indicators. A clinical model (C Model) was constructed using clinical indicators, and a combined model using clinical, tumor, and liver parenchyma indicators (C-T&L Model). We also compared their discrimination and calibration with that of the T&L Model. Discrimination was evaluated using a receiver operating characteristic (ROC) curve, and the area under the curve (AUC) was compared using the Delong test. Calibration was assessed using a calibration plot. Decision curve analysis (DCA) illustrated the net benefit of the optimal model. Finally, we performed subgroup analyses of the optimal model to determine whether its performance was affected by clinical factors. Patients were then divided into high- and low-risk populations using the median risk score in the training set, and their PFS was compared using Kaplan − Meier plots and Log-rank tests. An applet was designed to facilitate clinical applications.

All statistical analyses were performed using Python (version 3.8). A *p*-value < 0.05 was considered statistically significant.

## Results

### Baseline characteristics of the patients

A total of 422 patients were included in this study and divided into training (*n* = 303, from YPH, ZCPH and SPH) and external validation (*n* = 119, from ZPH, NFH and SAHG) datasets. During the initial treatment phase, 125 patients underwent liver resection (training dataset, *n* = 93; validation dataset, *n* = 32), and 297 patients underwent TACE (training dataset, *n* = 210; validation dataset, *n* = 87). During the follow-up period, 220 patients developed PD within 12 months (training dataset: *n* = 155; validation dataset: *n* = 65), and 56 patients died (training dataset: *n* = 45; validation dataset: *n* = 11). Except sex, hepatitis B infection, Child–Pugh grade, and ALT levels, no other difference were significant between training and validation datasets (Table 1).

### Necessity of synergistic tumor-liver parenchyma analysis

In total, 1,231 tumor-related indicators (eight morphological factors and 1,223 high-dimensional features) and 1,232 liver parenchyma-related indicators (nine morphological factors and 1,223 high-dimensional features) were extracted. After LASSO regression, 11 tumor indicators and 13 liver parenchyma indicators related to prognosis were identified (Supplementary Table 3, Supplementary Fig. 1). Utilizing these indicators, we constructed a tumor model (T Model) and a tumor-liver parenchyma model (T&L Model) to explore whether the liver parenchyma provided additional information for HCC prognosis prediction.

Regarding discrimination, the T&L Model outperformed the T Model at both 6 months (training: 0.747 vs. 0.722, Supplementary Fig. 2-a; validation: 0.719 vs. 0.683, Supplementary Fig. 2-c) and 12 months (training: 0.711 vs. 0.678, Supplementary Fig. 2-e; validation: 0.737 vs. 0.683, Supplementary Fig. 2-g), with the Delong test results displayed in Supplementary Table 4. Concerning calibration, the T&L Model outperformed the T Model (Supplementary Fig. 2-b, 2-d, 2-f, and 2-h). These results demonstrated that synergistic tumor-liver parenchyma analysis was reasonable and informative.

### Necessity of combining clinical and tumor-liver parenchyma information

Nine clinical indicators were included in the preliminary screen. LASSO regression was used for feature screening, and four clinical factors related to prognosis were identified: AFP level, Child–Pugh grade, BCLC stage, and treatment (Supplementary Table 3, Supplementary Fig. 1). Subsequently, we constructed a clinical model (C Model) and a combined model (C-T&L Model) as described above. The C Model, T&L Model, and C-T&L Model were compared to verify the necessity of combining clinical factors with tumor and liver parenchyma information.

**Fig. 4:**
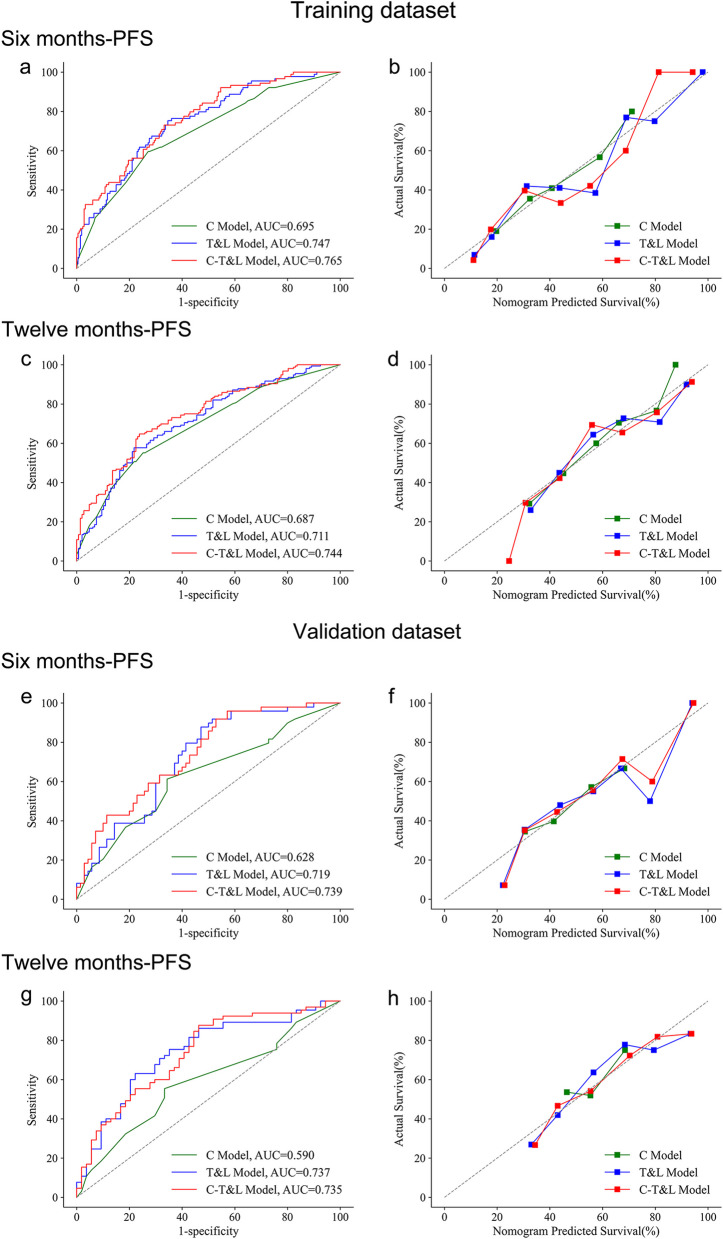
Comparison between the C Model, T&L Model, and C-T&L Model. Comparison between the C Model, T&L Model and C-T&L Model. Discrimination (with ROC curve) and calibration abilities are compared at both 6 months (**a**, **b**, **e**, **f**) and 12 months (**c**, **d**, **g**, **h**). In general, the C-T&L Model exhibited the best performance, especially for the AUC

**Fig. 5:**
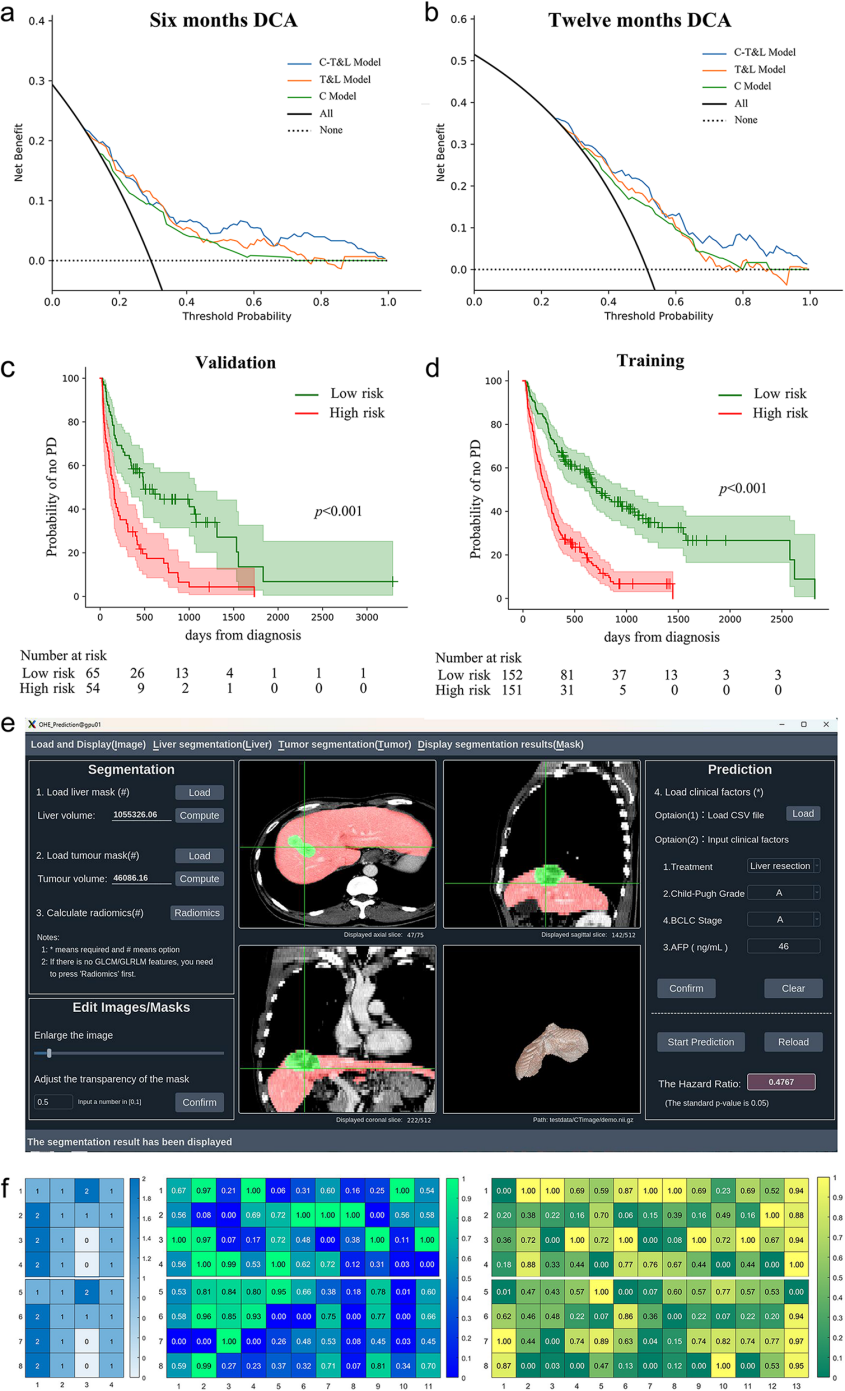
Decision curve, survival analysis, applet, and examples for C-T&L Model. Decision curve analysis (DCA) for C Model, T&L Model and C-T&L Model at (**a**) 6 months and (**b**) 12 months. Subdivided by a median risk score of 1.06, the high- and low-risk populations show significant differences in progression-free survival, both in the (**c**) training and (**d**) validation datasets. An applet (**e**) is constructed for the C-T&L Model, with eight patients (four pairs) displayed (**f**)

Regarding discrimination, the AUC of the C-T&L Model on the training dataset for 6 months and 12 months PFS was better than that of C Model and T&L Model (6 months: 0.765 vs. 0.695 vs. 0.747, Fig. 4-a; 12 months: 0.744 vs. 0.687 vs. 0.711; Fig. 4-c). The results were similar in the validation dataset (6 months: 0.739, 0.628, and 0.719, Fig. 4-e; 12 months: 0.735, 0.590, and 0.737; Fig. 4-g). Statistical differences were determined using the Delong test (Supplementary Table 4). Regarding calibration, the C-T&L Model outperformed C Model and T&L Model in both 6 (Fig. 4-b, f) and 12 months (Fig. 4-d, h). These results emphasize the contribution of tumor and liver parenchyma indicators to the improvement of model performance. The C-T&L Model was identified as the optimal model, and the DCA curve of 6 months and 12 months is shown in Fig. 5-a, b.

### Subgroup and survival analyses

Subgroup analyses based on AFP level, BCLC stage (0, A, or B), Child–Pugh grade (A or B), and treatment style (liver resection or TACE) showed that the performance of the C-T&L Model had similar performance in different subgroups. For 6 months, the result showed no statistical difference between groups in either the training dataset (AFP level: *p* = 0.245; BCLC stage:* p* = 0.452; Child–Pugh grade:* p* = 0.313; treatment style: *p* = 0.398, Supplementary Fig. 4-a, 4-b, 4-c and 4-d) or the validation dataset (AFP level: *p* = 0.294; BCLC stage:* p* = 0.638; Child–Pugh grade:* p* = 0.366; treatment style: *p* = 0.505, Supplementary Fig. 4-e, 4-f, 4-g and 4-h). In addition, the result was similar in 12 months (training dataset: 0.357, 0.433, 0.829, and 0.845, Supplementary Fig. 4-i, 4-j, 4-k and 4-l; validation dataset: 0.937, 0.402, 0.524, and 0.365, Supplementary Fig. 4-m, 4-n, 4-o and 4-p).

For survival analyses, patients were divided by the median risk score of training dataset set at 1.06 into low-risk population (risk score ≤ 1.06) and high-risk population (risk score > 1.06). The low-risk population had a longer PFS than the high-risk population, both in training dataset (median: 594 vs. 227 days; hazard ratio [HR] = 2.77; 95% confidence interval [CI]: 2.12–3.61; *p* < 0.001; Fig. 5-c) and validation dataset (median: 441 vs. 155 days; HR = 2.48; 95% CI: 1.65–3.72; *p* < 0.001; Fig. 5-d). The applet can be accessed at https://github.com/FuSirui123/Tumor-and-Liver-parenchyma-in-predicting-prognosis-of-HCC (Fig. 5-e), with eight examples (four pairs), as shown in (Fig. 5-f).

## Discussion

In this multicenter study, in addition to traditional clinical factors, 3D synergistic tumor-liver parenchyma analysis improved the efficacy prediction of HCC. The results indicated besides intratumor area, extra-tumor liver parenchyma area should also be studied, offering inspiration for similar researches.

HCC exhibits high metastasis and recurrence rates post-treatment, making the construction of prognostic models a longstanding focus. Traditionally, model construction mainly involved clinical and tumor indicators, including Child–Pugh grade, BCLC stage, and AFP [20–22]. However, in addition to these, the spread of HCC is influenced by the liver parenchyma, an aspect that is often overlooked. Recently, studies have proven that information from liver parenchyma can be used to predict to HCC occurrence and treatment efficacy [23, 24]. However, rather than single parameter such as stiffness, multi-dimensional analysis should be performed to extract information from the liver parenchyma. Therefore, based on 3D automatic segmentation, we used indicators within and beyond human visual system to assess change in liver parenchyma. Then by combining the information with clinical and tumorous indicators, we developed a more precise model.

For data mining of tumor and liver parenchyma, we used two types of indicators. The first type is represented by morphological indicators, which aims to quantify changes within human visual system. In our study, morphological indicators are volume or CT attenuation related. In morphological related indicators, for absolute tumor and liver parenchyma volume, body shape may introduce bias. Thus, we use self-control method, including the maximum diameter of the portal vein, as well as volume ratio between tumor and liver parenchyma. For CT attenuation related indicators, we emphasize the measurement of attenuation heterogeneity within the region of interest, which can be used to reflect heterogeneity [25]. Similarly, we also used parameters involving interquartile to control potential bias. The second type includes high-dimensional indicators measured by radiomic features [26], such as gray-level co-occurrence matrix and wavelet, etc. These indicators mainly focus on subtle changes beyond traditional human visual system.

As expected, compared to a model merely including tumor indicators, T&L Model had better performance, especially in calibration. This indicates the necessity of synergistic tumor and liver parenchyma analysis. Meanwhile, compared with the T&L Model, C-T&L Model had better performance, although the advantage was only significant in training dataset by 12 months, it still showed that combination of clinical, tumor, and liver parenchyma was reasonable to increase the accuracy of efficacy prediction.

Regarding the indicators included in the C-T&L Model, clinical factors include AFP level, Child–Pugh grade, BCLC stage, and treatment, which are generally recognized as prognostic factors at present and have been confirmed in previous studies [4, 20, 27]. For tumorous indicators, in addition to some high-dimensional factors, median CT attenuation is also identified. The results indicate that for HCC, blood supply (hypovascular vs. hypervascular) have more relationship with treatment efficacy [28, 29]. For liver parenchyma indicators, rather than tumor volume factors, the volume ratio between tumor and liver parenchyma is identified. The result manifests that the interaction between tumor and liver parenchyma is more important than simple tumor measurement. The lower value indicates that the liver parenchyma had an increased ability to limit tumor metabasis. Similarly, the identified radiomic features also reflect the difference in the liver parenchyma, which correlates to subtle changes in signal intensity of adjacent pixels/voxels or groups of pixels or voxels in CT images [30, 31]. Therefore, they may also reflect the ability of liver parenchyma in restricting metastasis such as formatting tumor capsule [32, 33].

Despite its strengths, this study has some limitations. First, due to the retrospective nature, it excluded patients undergoing ablation therapy and lacked samples from cohorts in other countries. Therefore, additional samples are required to further test the model’s generalizability. Second, to control bias, relatively strict inclusion criteria were used in this study, which resulted in a relatively small sample size and thereafter some unsolved issue (such as increased AUC but with insignificant *p*-value between T&L and T Model. Future studies should include larger sample sizes to obtain more comprehensive and detailed conclusions for the aspects. Third, since further liver segmentation was not performed, the entire liver parenchyma was taken as the research object. Differentiation analysis of individual liver segments (e.g., segments with tumors vs. without tumors) should be performed in the future. Fourth, for multiple lesions, due to technology limitation, we could only perform automatic segmentation for the target lesion, which was in consistence with the mRECIST criteria [17]. However, in future studies, more detailed analysis for lesions apart from target lesion should be explored. Fifth, to better assess liver fibrosis and cirrhosis, Magnetic Resonance Elastography (MRE), an early diagnostic test, can be used.

## Conclusion

In this study, 3D synergistic tumor-liver parenchyma analysis proved to provide additional insights into the prediction of HCC treatment efficacy. The methodologies used in this study may inspire further research on predicting HCC prognosis.

## Supplementary Information

Additional file 1. Supplementary material of 3D synergistic tumor-liver analysis further improves the efficacy prediction in hepatocellular carcinoma: a multi-center study.

## Data Availability

Due to the privacy of patients, the data related to patients cannot be available for public access but can be obtained from the corresponding author on reasonable request approved by the institutional review board of Zhuhai People’s Hospital.

## Abbreviations

AUC: Area Under the curve
BCLC: Barcelona clinic liver cancer
CT: Computed tomography
DCA: Decision curve analysis
HCC: Hepatocellular carcinoma
TACE: Transcatheter arterial chemoembolization
MRI: Magnetic resonance imaging
NLR: Neutrophil-to-lymphocyte ratio
PD: Progressive disease
PFS: Progression-free survival
ROC: Receiver operating characteristic curve
