# Supplementary material for: 3D synergistic tumor-liver analysis further improves the efficacy prediction in hepatocellular carcinoma: a multi-center study
Source: BMC Cancer. 2025 Jan 21;25:108. doi: 10.1186/s12885-025-13501-9 (PMC11748843; doi:10.1186/s12885-025-13501-9)
Supplement: Supplementary file 1 — Additional file 1. Supplementary material of 3D synergistic tumor-liver analysis further improves the efficacy prediction in hepatocellular carcinoma: a multi-center study. [file 12885_2025_13501_MOESM1_ESM.pdf]

Title: 3D synergistic tumor-liver analysis further improves the efficacy prediction in hepatocellular carcinoma: a multi-center study

Yurong Jiang; Jiawei Zhang; Zhaochen Liu; Jinxiong Zhang; Xiangrong Yu; Danyan Lin; Dandan Dong; Mingyue Cai; Chongyang Duan; Shuyi Liu; Wenhui Wang; Yuan Chen; Qiyang Li; Weiguo Xu; Meiyan Huang; Sirui Fu

Supplementary material

1. Supplementary Text 1. nnU-Net for segmentation
2. Supplementary Table 1. The major parameters of CT image acquisition
3. Supplementary Table 2. Candidate morphological factors of tumor and liver parenchyma
4. Supplementary Table 3. Identified factors for model construction
5. Supplementary Table 4. Delong test of the model comparison
6. Supplementary Fig. 1. Hazard ratios and 95%CI of identified factors
7. Supplementary Fig. 2. Comparison between the T Model and T&L Model
8. Supplementary Fig. 3. Box chart of three prediction models
9. Supplementary Fig. 4. Subgroup analysis of C-T&L Model

1. Supplementary Text 1. nnU-Net for segmentation

In this study, venous-phase 3D CT images were used as input data. The original image size was (144, 512, 512), and during preprocessing, the voxel spacing was resampled to (1.5, 0.6470, 0.6470). The images were standardized using Z-score normalization. The network architecture was based on the 3D U-Net under the nnU-Net framework (version 1.7.0), with an input patch size of (48, 192, 192). This patch size was chosen to accommodate GPU memory limitations, and the batch size was set to 2. The experiments were conducted on a single NVIDIA 2080Ti GPU with 12GB of memory, using Python 3.8 and PyTorch 1.11.0.

Data augmentation was performed using the default nnU-Net strategies, including but not limited to random flipping, rotation, and noise addition, to improve the model's generalization ability. The initial learning rate was set to 0.01 and decreased progressively over the course of 1000 training epochs. The loss function was a weighted combination of Dice loss and Cross-Entropy loss, aimed at optimizing segmentation tasks for the liver and liver tumors.

The segmentation targets included the liver and liver tumors, with labels defined as 0, 1, and 2, where 0 represents the background, 1 represents the liver, and 2 represents the tumor. During the post-processing stage, for tumor segmentation, only the largest tumor by volume was retained. This decision was based on clinical relevance, as the tumor with the highest burden has the greatest impact on the patient's prognosis. Additionally, any holes within the segmentation targets 1 (liver) and 2 (tumor) were filled to refine the segmentation results.

The evaluation metrics included the Dice Similarity Coefficient (DSC) and the 95% Hausdorff Distance (HD95), which were used to assess the overlap and boundary matching of the segmentation results, respectively, providing a comprehensive evaluation of the model's performance in liver and liver tumor segmentation tasks.

2. Supplementary Table 1. The major parameters of CT image acquisition

Hospitals	Scanner	TV (kV)	TC (mA)	RT (s)	DC (mm)	FOV (mm)	PM	Reconstruction	ST (mm)
**NFH**	Philips Brilliance	120	142	0.75	128×0.625	300×300	512×512	Filter sharp (C)	5 mm
**SPH**	Philips Brilliance	120	250	0.50	64×0.625	500×500	1024×1024	Filter sharp (C)	5 mm
**ZCPH**	Philips Brilliance	120	250	0.50	128×0.625	350×350	512×512	Filter standard (B)	5 mm
**YPH**	Philips Brilliance	120	300	0.75	64×0.625	350×350	512×512	Filter sharp (C)	2mm
**SAHG**	Philips Brilliance	120	250	0.50	128×0.625	350×350	512×512	Filter standard (B)	5 mm
**ZPH**	Siemens Somatom Definition Flash	120	160	0.50	64×0.625	350×350	512×512	Filter sharp (C)	2 mm<br>5 mm

TV: tube voltage; TC: tube current; RT: rotation time; DC: detector collimation; FOV: field of view; PM: pixel matrix; ST: slice thickness; NFH: Nanfang Hospital; SPH: Shenzhen People's Hospital; ZCPH: Zhongshan City People's Hospital; YPH: Yang-jiang People's Hospital; SAHG: The Second Affiliated Hospital of Guangzhou Medical University; ZPH: Zhuhai People's Hospital.

3. Supplementary Table 2. Candidate morphological factors of tumor and liver parenchyma

Classification	Factor name
**Tumor**	
**Volume**	Tumor volume
**CT attenuation**	Mean CT attenuation of the tumor
	Median CT attenuation of the tumor
	25th percentile of the CT attenuation of the tumor
	75th percentile of the CT attenuation of the tumor
	Interquartile CT attenuation of the tumor
	CT attenuation of the tumor at a rate of 25%
	CT attenuation of the tumor at a rate of 75%
**Liver parenchyma**	
**Volume**	Ratio between the liver parenchyma volume and the maximum diameter of the portal vein
	Ratio between the tumor volume and the liver parenchyma volume
**CT attenuation**	Mean CT attenuation of the liver parenchyma
	Median CT attenuation of the liver parenchyma
	25th percentile of the CT attenuation of the liver parenchyma
	75th percentile of the CT attenuation of the liver parenchyma
	Interquartile CT attenuation of the liver parenchyma
	CT attenuation of the liver parenchyma at a rate of 25%
	CT attenuation of the liver parenchyma at a rate of 75%

CT: computed tomography

4. Supplementary Table 3. Identified factors for model construction

Classification	Factor
**Clinical factors**	
	AFP level
	Child-Pugh grade
	BCLC stage
	Treatment style
**Tumor features**	
**Morphological**	Median CT attenuation of the tumor
**High-dimensional**	Tumor_original_glcm_MaximumProbability
	Tumor_wavelet-HHL_glrlm_ShortRunHighGrayLevelEmphasis
	Tumor_wavelet-HHL_gldm_DependenceEntropy
	Tumor_wavelet-HHL_glcm_ClusterShade
	Tumor_wavelet-HLH_glcm_ClusterShade
	Tumor_wavelet-LHL_glcm_ClusterShade
	Tumor_log-sigma-3-mm-3D_glcm_ClusterShade
	Tumor_log-sigma-1-mm-3D_gldm_DependenceVariance
	Tumor_log-sigma-1-mm-3D_firstorder_Skewness
	Tumor_log-sigma-1-mm-3D_glszm_GrayLevelNonUniformityNormalized
**Liver parenchyma features**	
**Morphological**	Ratio between the tumor volume and the liver parenchyma volume
**High-dimensional**	Rliver_original_shape_Sphericity
	Rliver_original_shape_MajorAxisLength
	Rliver_original_shape_Maximum2DDiameterColumn
	Rliver_original_glcm_MaximumProbability
	Rliver_gradient_glrlm_LongRunHighGrayLevelEmphasis
	Rliver_gradient_glrlm_LongRunEmphasis
	Rliver_gradient_glrlm_LongRunLowGrayLevelEmphasis
	Rliver_wavelet-HLL_glszm_ZoneEntropy
	Rliver_wavelet-HHL_glszm_SmallAreaLowGrayLevelEmphasis
	Rliver_wavelet-LHH_glszm_SmallAreaLowGrayLevelEmphasis
	Rliver_wavelet-LHH_glszm_GrayLevelNonUniformityNormalized
	Rliver_log-sigma-1-mm-3D_glszm_SmallAreaLowGrayLevelEmphasis

AFP: alpha-fetoprotein; BCLC: Barcelona Clinic Liver Cancer; CT: computed tomography

5. Supplementary Table 4. Delong test of the model comparison

	**Six months**	**One year**
**T Model vs. T&L Model**		
Training dataset	0.196	0.073
Validation dataset	0.253	0.103
**C Model vs. C-T&L Model**		
Training dataset	0.006*	0.020*
Validation dataset	0.008*	0.001*
**T&L Model vs. C-T&L Model**		
Training dataset	0.256	0.014*
Validation dataset	0.439	0.916

*p<0.050

6. Supplementary Fig. 1. Hazard ratios and 95%CI of Identified factors

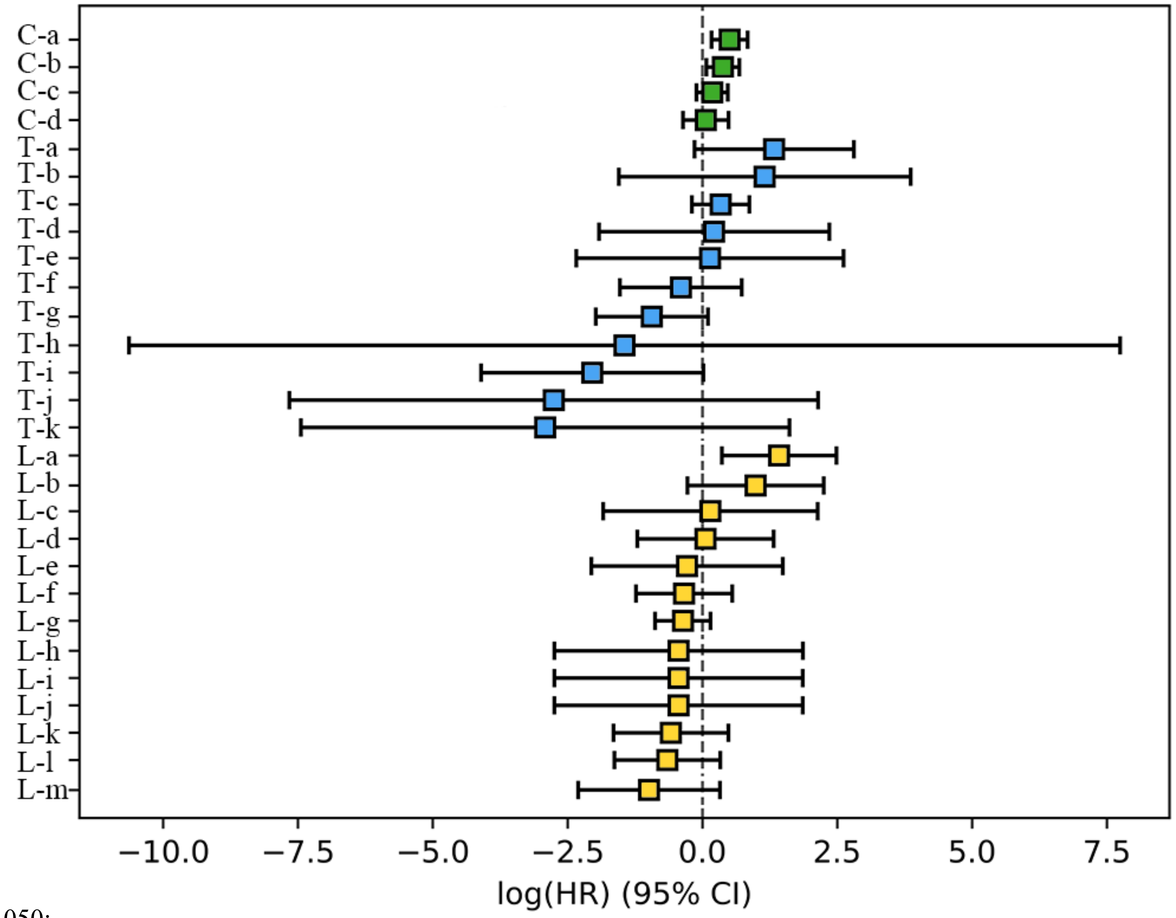

Factors	HR	95% confidence interval [CI]
**C-a**	1.65	1.18-2.31
**C-b**	1.46	1.08-1.98
**C-c**	1.20	0.90-1.60
**C-d**	1.06	0.70-1.62
**T-a**	3.78	0.86-16.53
**T-b**	3.17	0.21-47.46
**T-c**	1.40	0.82-2.39
**T-d**	1.24	0.15-10.50
**T-e**	1.15	0.10-13.70
**T-f**	0.67	0.22-2.07
**T-g**	0.39	0.14-1.11
**T-h**	0.24	0.00-2307.22
**T-i**	0.13	0.02-1.02
**T-j**	0.06	0.00-8.54
**T-k**	0.05	0.00-5.02
**L-a**	4.14	1.43-11.97
**L-b**	2.68	0.76-9.47
**L-c**	1.16	0.16-8.47
**L-d**	1.06	0.30-3.74
**L-e**	0.75	0.13-4.43
**L-f**	0.71	0.29-1.73
**L-g**	0.70	0.42-1.16
**L-h**	0.64	0.06-6.43
**L-i**	0.64	0.06-6.43
**L-j**	0.64	0.06-6.43
**L-k**	0.56	0.19-1.61
**L-l**	0.52	0.20-1.39
**L-m**	0.37	0.10-1.38

* *p*<0.050;

C-a: Treatment style; C-b: BCLC stage; C-c: AFP level; C-d: Child-Pugh grade; T-a: Tumor_log-sigma-1-mm-3D_firstorder_Skewness; T-b: Tumor_log-sigma-3-mm-3D_glcm_ClusterShade; T-c: Tumor_original_glcm_MaximumProbability; T-d: Tumor_wavelet-HLH_glcm_ClusterShade; T-e: Tumor_wavelet-HHL_gldm_DependenceEntropy; T-f: Tumor_log-sigma-1-mm-3D_glszm_GrayLevelNonUniformityNormalized; T-g: Median CT attenuation of the tumor; T-h: Tumor_wavelet-LHL_glcm_ClusterShade; T-i: Tumor_log-sigma-1-mm-3D_gldm_DependenceVariance; T-j: Tumor_wavelet-HHL_glrlm_ShortRunHighGrayLevelEmphasis; T-k: Tumor_wavelet-HHL_glcm_ClusterShade; L-a: Rliver_original_shape_Maximum2DDiameterColumn; L-b: Rliver_wavelet-LHH_glszm_SmallAreaLowGrayLevelEmphasis; L-c: Rliver_original_shape_Sphericity; L-d: Rliver_wavelet-HLL_glszm_ZoneEntropy; L-e: Ratio between the tumor volume and the liver parenchyma volume; L-f: Rliver_log-sigma-1-mm-3D_glszm_SmallAreaLowGrayLevelEmphasis; L-g: Rliver_original_glcm_MaximumProbability; L-h: Rliver_gradient_glrlm_LongRunHighGrayLevelEmphasis; L-i: Rliver_gradient_glrlm_LongRunEmphasis; L-j: Rliver_gradient_glrlm_LongRunLowGrayLevelEmphasis; L-k: Rliver_original_shape_MajorAxisLength; L-l: Rliver_wavelet-HHL_glszm_SmallAreaLowGrayLevelEmphasis; L-m: Rliver_wavelet-LHH_glszm_GrayLevelNonUniformityNormalized.

7. Supplementary Fig. 2. Comparison between the T Model and T&L Model

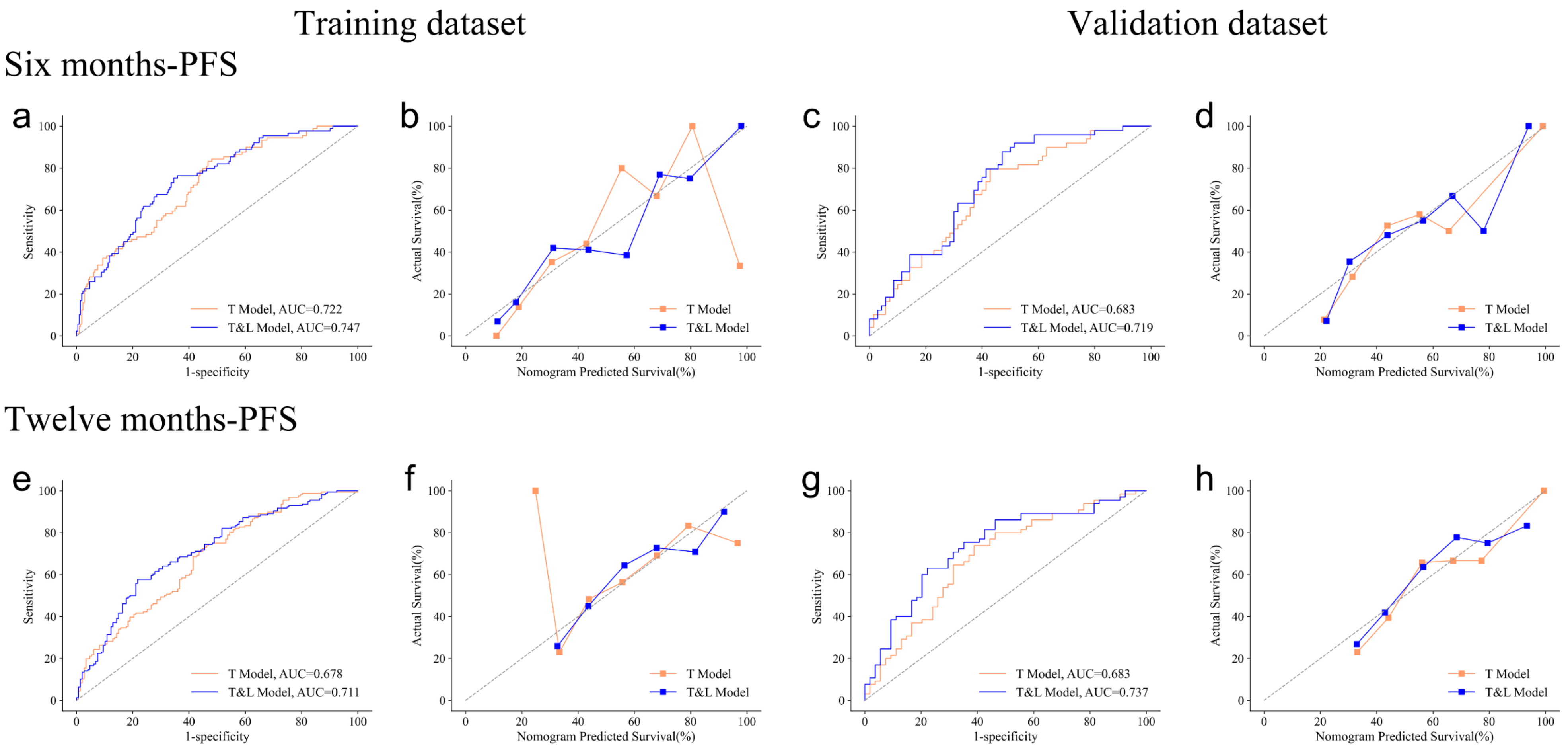

Comparison between the T Model and T&L Model. The discrimination (with ROC curve) and calibration are compared at both 6-months (a, b, c, d) and 12-months (e, f, g, h). The T&L Model performs better than the T Model, especially in terms of the AUC.

8. Supplementary Fig. 3. Box chart of three prediction models

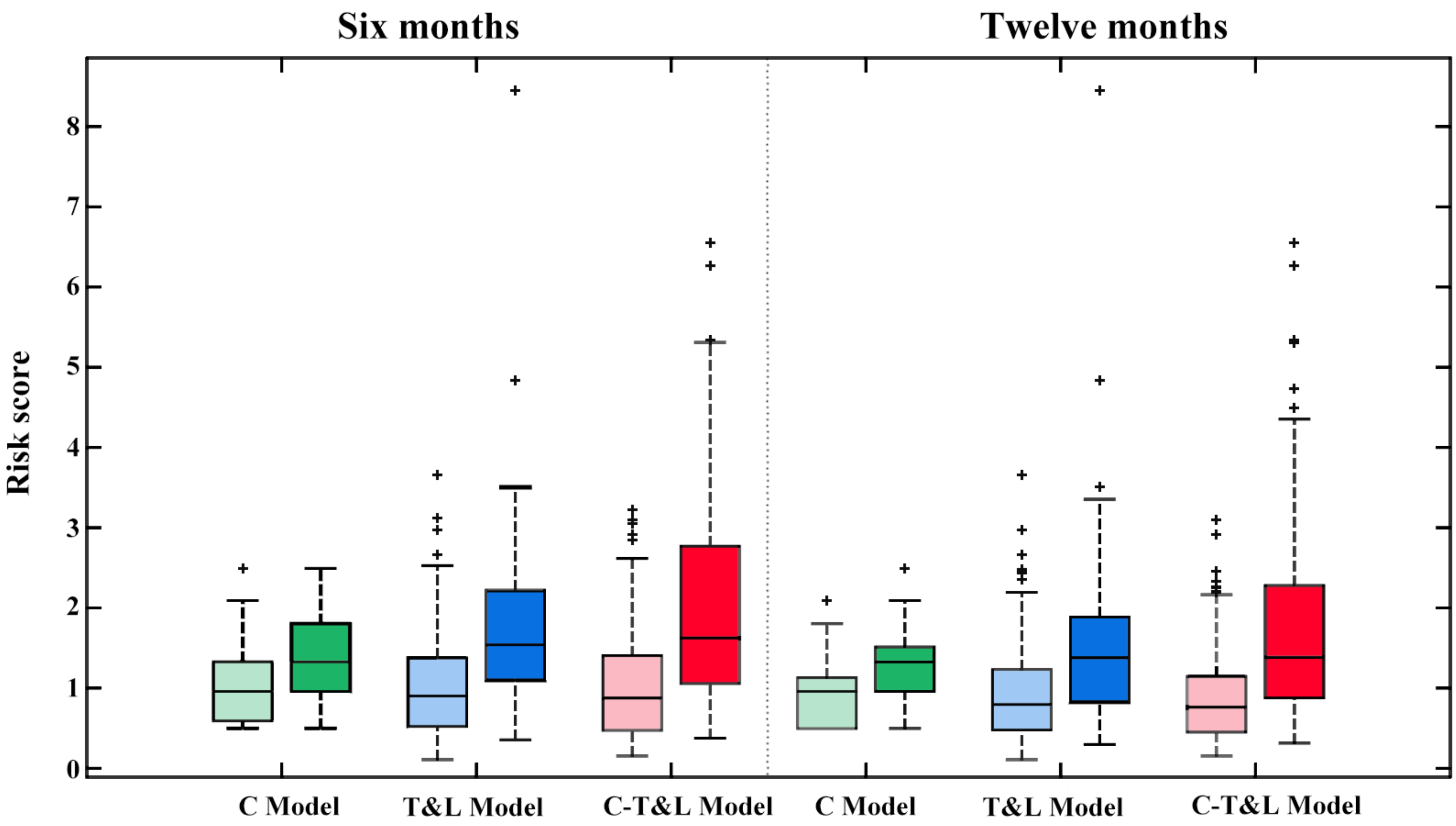

The study population is divided into two cohorts based on the presence of PD within 6 or 12 months.

Risk scores of the C Model, T&L Model, and C-T&L Model for the cohort that develops PD within 6 or 12 months. The risk scores of the three models are increased, which is consistent with the fact that the risk value of the cohort was high.

Risk scores of the C Model, T&L Model, and C-T&L Model for the cohort without PD within 6 or 12 months. The risk scores of the three models are decreased, which is consistent with the fact that the risk value of the cohort was low. PD, progressive disease.

9. Supplementary Fig. 4. Subgroup analysis of C-T&L Model

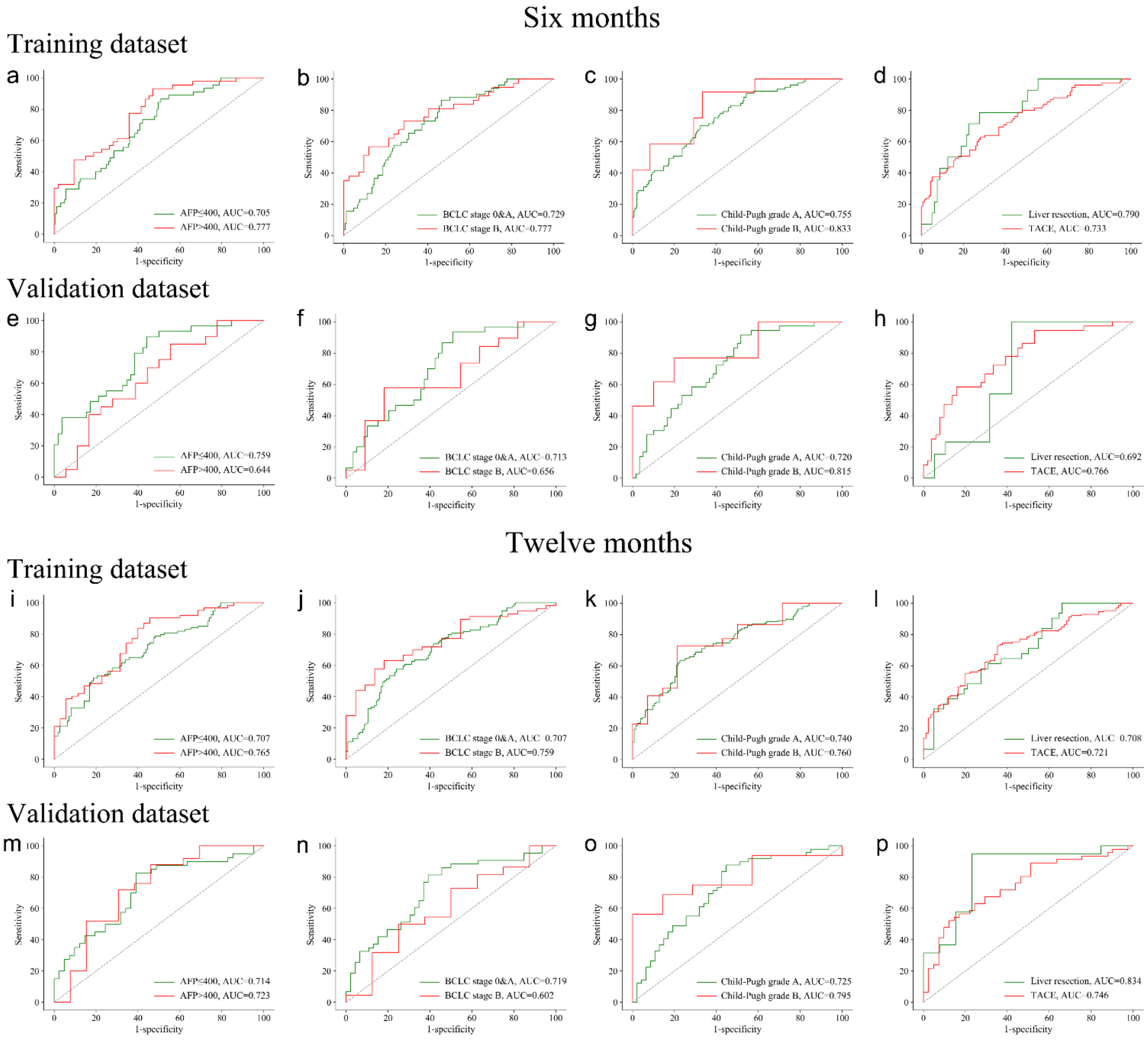

The performance of the integrated model (C-T&L Model) is not influenced by AFP level (a, e, i, m), BCLC stage (b, f, j, n), Child–Pugh grade (c, g, k, o), or treatment style (d, h, l, p). AFP: alpha-fetoprotein; BCLC: Barcelona Clinic Liver Cancer
